# 3-Chloro-4-dimethyl­amino-5-[(1*R*,2*S*,5*R*)-2-isopropyl-5-methyl­cyclo­hex­yloxy]furan-2(5*H*)-one

**DOI:** 10.1107/S1600536811015583

**Published:** 2011-05-20

**Authors:** Xiu-Mei Song, Ning Wang, Zhao-Yang Wang, Zong-Cai Feng

**Affiliations:** a Research Center of Chemistry & Materials, Zhanjiang Normal College, People’s Republic of China; b Development Center for New Materials Engineering &, Technology in Universities of Guangdong, People’s Republic of China; c Chemistry Science & Technology School, Zhanjiang Normal College, Zhanjiang 524048, People’s Republic of China; d School of Chemistry and Environment, South China Normal University, Guangzhou 510006, People’s Republic of China; e Research Institute of Tsinghua University in Shenzhen, Shenzhen 518055, People’s Republic of China

## Abstract

The title compound, C_16_H_26_ClNO_3_ contains one almost planar furan­one ring [maximum deviation of 0.021 (2) Å for the O atom] with a stereogenic center (*S*) and one cyclo­hexane ring which displays a chair conformation and has three stereogenic centers [*S* at the C atom bearing the isopropyl group, *R* at the C atom attached to the O atom and *R* at the C atom bearing the methyl group].

## Related literature

For natural products containing a 2(5*H*)-furan­one subunit, see: Ming *et al.* (2002 ▶). For biologically active 2(5*H*)-furan­ones, see: Bailly *et al.* (2008 ▶). For the synthesis of 2(5*H*)-furan­ones with substituents in positions 3 and 4, see: Van Oeveren *et al.* (1994 ▶); For related structures, see: Chen *et al.* (1995 ▶); Martín & Mateo (1995 ▶); Gawronski *et al.* (1997 ▶). For the use of benzimidazoles in organic synthesis, see: Mao *et al.* (2010 ▶). For standard bond lengths, see: Allen *et al.* (1987 ▶); Orpen *et al.* (1989 ▶). For the structures of heterosubstituted 2(5*H*)-furan­ones, see: Gawronski *et al.* (1997 ▶). For the synthesis and structure of optically pure 5-(*l*-menth­yloxy)-3,4-dichloro-2(5*H*)-furan­one, see: Chen & Geng (1993 ▶). For a description of the Cambridge Structural Database, see: Allen (2002 ▶).
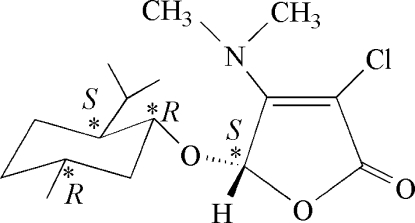


## Experimental

### 

#### Crystal data

C_16_H_26_ClNO_3_
*M* *_r_* = 315.83
Orthorhombic, 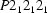

*a* = 7.5438 (5) Å
*b* = 8.1631 (5) Å
*c* = 28.5953 (17) Å
*V* = 1760.92 (19) Å^3^
*Z* = 4
Mo *K*α radiation
μ = 0.23 mm^−1^
*T* = 296 K
0.23 × 0.22 × 0.19 mm

#### Data collection

Bruker APEXII area-detector diffractometer
Absorption correction: multi-scan (*SADABS*; Sheldrick, 1996 ▶) *T*_min_ = 0.950, *T*_max_ = 0.958
9112 measured reflections
2922 independent reflections
1951 reflections with *I* > 2σ(*I*)
*R*_int_ = 0.049

#### Refinement

*R*[*F* ^2^ > 2σ(*F* ^2^)] = 0.047
*wR*(*F* ^2^) = 0.117
*S* = 1.02
2922 reflections
196 parameters
H-atom parameters constrained
Δρ_max_ = 0.15 e Å^−3^
Δρ_min_ = −0.14 e Å^−3^
Absolute structure: Flack (1983 ▶), 1066 Friedel pairs
Flack parameter: −0.05 (9)

### 

Data collection: *APEX2* (Bruker, 2004 ▶); cell refinement: *SAINT* (Bruker, 2004 ▶); data reduction: *SAINT*; program(s) used to solve structure: *SHELXS97* (Sheldrick, 2008 ▶); program(s) used to refine structure: *SHELXL97* (Sheldrick, 2008 ▶); molecular graphics: *SHELXTL* (Sheldrick, 2008 ▶); software used to prepare material for publication: *SHELXL97*.

## Supplementary Material

Crystal structure: contains datablocks I, global. DOI: 10.1107/S1600536811015583/si2350sup1.cif

Structure factors: contains datablocks I. DOI: 10.1107/S1600536811015583/si2350Isup2.hkl

Supplementary material file. DOI: 10.1107/S1600536811015583/si2350Isup3.cml

Additional supplementary materials:  crystallographic information; 3D view; checkCIF report

## Figures and Tables

**Fig. 1.**
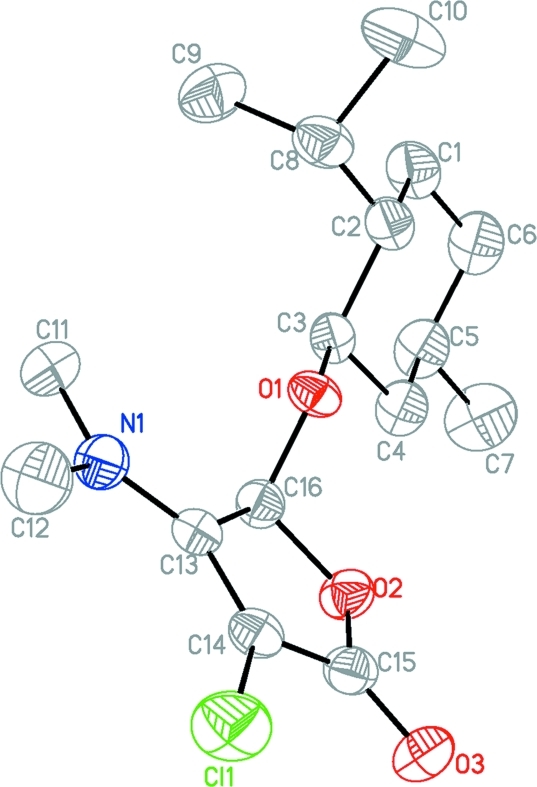
The molecular structure of the title compound. Displacement ellipsoids drawn at the 30% probability level, hydrogen atoms have been omitted for clarity.

## supplementary crystallographic information

### Comment

2(5*H*)-Furanones, also known as crotonolactones or butenolides, have attracted increasing attention of many organic chemists due to their presence as a subunit in many natural products (Ming *et al.*, 2002). This core unit is the key structure to induce a variety of biological phenomena like antifungal, anti-inflamatory, antibacterial, and HIV-1 anti-integrase (Bailly *et al.*, 2008). In recent years, chiral 2(5*H*)-furanones with substituents in positions 3 and 4 have been synthesized in several laboratories (Van Oeveren *et al.*, 1994) and their crystal structures have been reported (Chen *et al.*, 1995; Martín & Mateo, 1995; Gawronski *et al.*, 1997).

Meanwhile, benzimidazoles were widely used in various areas, especially serving as key intermediate in organic synthesis (Mao *et al.*, 2010). In our initial study, we focused on the Michael addition-elimination reaction of 3,4-dichloro-5-(*S*)-(*l*-menthyloxy)-2(5*H*)-furanone and nucleophilic reagent imidazole. On the basis of the experiment, no target molecule was obtained, but the unexpected product, 3-chloro-4-*N*,*N*-dimethyl-5-(*S*)-(*l*-menthyloxy)-2(5*H*)-furanone that came from reaction of 3,4-dichloro-5-(*S*)-(*l*-menthyloxy)-2(5*H*)-furanone and the solvent *N*,*N*-dimethylformamide was given.

In the title compound (Fig. 1), the cyclohexane ring displays a chair conformation and has three stereogenic centers (C2(*S*), C3(*R*), C5(*R*)), and the planar core structure subunit, the furanone ring exhibits a C16(*S*) center with a maximum deviation of 0.021 (2) Å of O2 from the mean plane of the five atoms defining the plane, r.m.s deviation is 0.0158 Å.

Cl substitution at C14 causes significant torsion around the C13—N1 bond. The C14—C13—N1—C12 torsion angle amounts to 5.2 (5)°, this indicates a significant twist around the C13—N1 bond resulting from Cl substitution at C14.

At the same time, N1 is only 0.034 (5) Å from the furanone ring plane. This is one manifestation of the extensive conjugation of the N1 lone pair, the C13═C14 double bond and the C15 carbonyl bond. The geometrical consequence is a shortening of the N1—C13 bond to an value of 1.331 (3) Å, which might be compared with the average value of 1.355 (14) Å for the C—N bond in C═C—N—(C)_2_ system (Orpen *et al.*, 1989; Gawronski *et al.*, 1997). The shortening of the N1—C13 bond is accompanied by the lengthening of the formally C13═C14 double bond to an value of 1.354 (3) Å, as compared with the value of 1.323 (13) Å quoted for cyclopentene and with 1.340 (13) Å in conjugated systems (Orpen *et al.*, 1989).

And the most striking geometrical change due to conjugation is found in the furanone ring. Comparison with the non-fused furanones which contain oxygen function at C(4) (22 observations subtracted from the Cambridge Structural Database 14) (Allen, 2002) reveals significant shortening of the formally single C(*sp*^2^)—C(*sp*^2^) bond to an value of 1.428 (4) Å and simultaneous lengthening of the *C*(carbonyl)—O bond to an value of 1.373 (3) Å. In non-fused furanones the two bonds have the mean values of 1.466 (26) Å and 1.362 (16) Å, respectively (Allen *et al.*, 1987; Gawronski *et al.*, 1997). This might indicate that the essential part of the electron delocalization is concentrated in the N1, C13, C14, C15 and O3 region, and takes place at the expense of delocalization within the ester function.

### Experimental

The optically pure precursor 3,4-dichloro-5-(*S*)-(*l*-menthyloxy)-2(5*H*)-furanone was prepared according to the literature procedure (Chen & Geng, 1993).

The title compond, 3-chloro-4-*N*,*N*-dimethyl-5-(*S*)-(*l*-menthyloxy)-2(5*H*)-furanone, was prepared by reaction of 3,4-dichloro-5-(*S*)-(*l*-menthyloxy)-2(5*H*)-furanone (3 mmol) and DMF (1 ml) at 80 °C, catalyzed by sodium ethanol (3 mmol) under N_2_ atmosphere. After stirring for 24 h, ice water was added to the mixture and then extracted by dichloromethane. The solvent was evaporated *in vacuo* and the precipitate was purified by silica gel column chromatography with gradient mixture of petroleum ether and ethyl acetate (Yield 21.3%). Single crystals of the title compound were obtained by slow evaporation of a solution in acetonitrile at room temperature.

Data for (I): m.p.161.0–163.0 °C; IR (KBr) ν: 2953.73, 748.40, 1630.61 cm^-1^; ^1^H NMR (400 MHz, CDCl_3_, TMS): 0.762 (3*H*, *d*, *J* = 6.8 Hz, CH_3_-7), 0.820–0.858 (1*H*, *m*, CH-8), 0.909 (3*H*, *s*, CH_3_-9), 0.926 (3*H*, *s*, CH_3_-10), 0.956–1.170 (2*H*, *m*, CH_2_-6), 1.290–1.427 (2*H*, *m*, CH-2, CH-5), 1.633–1.695 (2*H*, *m*, CH_2_-1), 2.171–2.211 (2*H*, *m*, CH_2_-4), 3.186 (6*H*, *s*, CH_3_-11, CH_3_-12), 3.532–3.845 (1*H*, *ddd*, *J* = 4.4 Hz, 4.4 Hz, 4.4 Hz, CH-3), 5.757 (1*H*, *s*, CH-16).

### Refinement

All H atoms were positioned in calculated positions (C—H = 0.96 Å or 0.97 Å or 0.98 Å) and were refined using a riding model, with *U*_iso_(H) = 1.2 *U*_eq_(C) for methylene or methine H atoms and *U*_iso_(H) = 1.5 *U*_eq_(C) for methyl H atoms.

### Crystal data

**Table:**

C_16_H_26_ClNO_3_	*F*(000) = 680
*M**_r_* = 315.83	*D*_x_ = 1.191 Mg m^−^^3^
Orthorhombic, *P*2_1_2_1_2_1_	Mo *K*α radiation, λ = 0.71073 Å
Hall symbol: P 2ac 2ab	Cell parameters from 3099 reflections
*a* = 7.5438 (5) Å	θ = 2.8–20.5°
*b* = 8.1631 (5) Å	µ = 0.23 mm^−^^1^
*c* = 28.5953 (17) Å	*T* = 296 K
*V* = 1760.92 (19) Å^3^	Block, colourless
*Z* = 4	0.23 × 0.22 × 0.19 mm

### Data collection

**Table:**

Bruker APEXII area-detector diffractometer	2922 independent reflections
Radiation source: fine-focus sealed tube	1951 reflections with *I* > 2σ(*I*)
graphite	*R*_int_ = 0.049
φ and ω scans	θ_max_ = 25.2°, θ_min_ = 1.4°
Absorption correction: multi-scan (*SADABS*; Sheldrick, 1996)	*h* = −8→7
*T*_min_ = 0.950, *T*_max_ = 0.958	*k* = −9→6
9112 measured reflections	*l* = −23→34

### Refinement

**Table:**

Refinement on *F*^2^	Hydrogen site location: inferred from neighbouring sites
Least-squares matrix: full	H-atom parameters constrained
*R*[*F*^2^ > 2σ(*F*^2^)] = 0.047	*w* = 1/[σ^2^(*F*_o_^2^) + (0.055*P*)^2^] where *P* = (*F*_o_^2^ + 2*F*_c_^2^)/3
*wR*(*F*^2^) = 0.117	(Δ/σ)_max_ < 0.001
*S* = 1.02	Δρ_max_ = 0.15 e Å^−^^3^
2922 reflections	Δρ_min_ = −0.14 e Å^−^^3^
196 parameters	Extinction correction: *SHELXL97* (Sheldrick, 2008), Fc^*^=kFc[1+0.001xFc^2^λ^3^/sin(2θ)]^-1/4^
0 restraints	Extinction coefficient: 0.016 (2)
Primary atom site location: structure-invariant direct methods	Absolute structure: Flack (1983), 1066 Friedel pairs
Secondary atom site location: difference Fourier map	Flack parameter: −0.05 (9)

### Special details

**Table:**

Geometry. All e.s.d.'s (except the e.s.d. in the dihedral angle between two l.s. planes) are estimated using the full covariance matrix. The cell e.s.d.'s are taken into account individually in the estimation of e.s.d.'s in distances, angles and torsion angles; correlations between e.s.d.'s in cell parameters are only used when they are defined by crystal symmetry. An approximate (isotropic) treatment of cell e.s.d.'s is used for estimating e.s.d.'s involving l.s. planes.
Refinement. Refinement of *F*^2^ against ALL reflections. The weighted *R*-factor *wR* and goodness of fit *S* are based on *F*^2^, conventional *R*-factors *R* are based on *F*, with *F* set to zero for negative *F*^2^. The threshold expression of *F*^2^ > σ(*F*^2^) is used only for calculating *R*-factors(gt) *etc*. and is not relevant to the choice of reflections for refinement. *R*-factors based on *F*^2^ are statistically about twice as large as those based on *F*, and *R*- factors based on ALL data will be even larger.

### Fractional atomic coordinates and isotropic or equivalent isotropic displacement parameters (Å^2^)

**Table:**

	*x*	*y*	*z*	*U*_iso_*/*U*_eq_	
C1	0.4580 (5)	0.8674 (4)	0.45480 (10)	0.0785 (11)	
H1A	0.3934	0.7827	0.4714	0.094*	
H1B	0.5740	0.8773	0.4691	0.094*	
C2	0.4806 (4)	0.8152 (4)	0.40327 (9)	0.0628 (9)	
H2	0.5491	0.9020	0.3880	0.075*	
C3	0.2995 (4)	0.8133 (3)	0.37990 (9)	0.0525 (8)	
H3	0.2272	0.7270	0.3942	0.063*	
C4	0.2051 (5)	0.9754 (3)	0.38525 (9)	0.0701 (10)	
H4A	0.0895	0.9685	0.3705	0.084*	
H4B	0.2724	1.0599	0.3693	0.084*	
C5	0.1815 (6)	1.0237 (4)	0.43669 (11)	0.0804 (11)	
H5	0.1112	0.9379	0.4519	0.096*	
C6	0.3610 (7)	1.0271 (4)	0.45991 (11)	0.0893 (12)	
H6A	0.4316	1.1140	0.4462	0.107*	
H6B	0.3462	1.0514	0.4929	0.107*	
C7	0.0799 (7)	1.1841 (5)	0.44127 (12)	0.1207 (16)	
H7A	0.0713	1.2137	0.4737	0.181*	
H7B	−0.0369	1.1710	0.4285	0.181*	
H7C	0.1413	1.2688	0.4245	0.181*	
C8	0.5869 (5)	0.6562 (4)	0.39706 (11)	0.0718 (10)	
H8	0.5775	0.6257	0.3640	0.086*	
C9	0.5161 (5)	0.5122 (4)	0.42518 (12)	0.0989 (14)	
H9A	0.5293	0.5345	0.4580	0.148*	
H9B	0.5814	0.4151	0.4173	0.148*	
H9C	0.3930	0.4960	0.4181	0.148*	
C10	0.7855 (5)	0.6823 (6)	0.40726 (13)	0.1098 (14)	
H10A	0.8276	0.7752	0.3899	0.165*	
H10B	0.8507	0.5865	0.3981	0.165*	
H10C	0.8019	0.7016	0.4401	0.165*	
C11	0.2476 (5)	0.4116 (4)	0.31317 (11)	0.0793 (11)	
H11A	0.3620	0.3966	0.3272	0.119*	
H11B	0.1923	0.3069	0.3087	0.119*	
H11C	0.1751	0.4777	0.3333	0.119*	
C12	0.3298 (6)	0.3891 (4)	0.23027 (11)	0.0928 (12)	
H12A	0.2355	0.3726	0.2082	0.139*	
H12B	0.3670	0.2853	0.2426	0.139*	
H12C	0.4280	0.4411	0.2149	0.139*	
C13	0.2303 (4)	0.6503 (3)	0.26159 (9)	0.0495 (7)	
C14	0.2256 (4)	0.7458 (3)	0.22286 (9)	0.0530 (8)	
C15	0.1758 (4)	0.9092 (4)	0.23489 (11)	0.0588 (8)	
C16	0.1844 (4)	0.7605 (3)	0.30258 (10)	0.0529 (8)	
H16	0.0818	0.7180	0.3198	0.063*	
Cl1	0.26385 (14)	0.70164 (12)	0.16516 (3)	0.0940 (4)	
N1	0.2678 (3)	0.4928 (3)	0.26825 (8)	0.0609 (7)	
O1	0.3317 (3)	0.7719 (2)	0.33142 (6)	0.0524 (5)	
O2	0.1456 (3)	0.9169 (2)	0.28218 (6)	0.0627 (6)	
O3	0.1584 (3)	1.0295 (3)	0.21086 (7)	0.0817 (7)	

### Atomic displacement parameters (Å^2^)

**Table:**

	*U*^11^	*U*^22^	*U*^33^	*U*^12^	*U*^13^	*U*^23^
C1	0.098 (3)	0.095 (3)	0.0424 (19)	−0.022 (2)	−0.0045 (19)	−0.0019 (18)
C2	0.075 (3)	0.077 (2)	0.0371 (18)	−0.022 (2)	−0.0020 (17)	0.0055 (15)
C3	0.062 (2)	0.0594 (16)	0.0362 (16)	−0.0128 (16)	0.0064 (15)	−0.0012 (12)
C4	0.095 (3)	0.0667 (18)	0.0485 (18)	−0.0030 (19)	0.0067 (19)	−0.0073 (14)
C5	0.116 (4)	0.075 (2)	0.050 (2)	0.001 (2)	0.010 (2)	−0.0140 (16)
C6	0.137 (4)	0.086 (2)	0.045 (2)	−0.017 (3)	0.002 (2)	−0.0115 (18)
C7	0.174 (4)	0.106 (3)	0.082 (3)	0.036 (3)	0.010 (3)	−0.038 (2)
C8	0.070 (3)	0.093 (3)	0.053 (2)	−0.005 (2)	−0.0094 (19)	0.0014 (18)
C9	0.118 (4)	0.096 (3)	0.083 (3)	0.003 (3)	−0.010 (3)	0.025 (2)
C10	0.069 (3)	0.178 (4)	0.083 (3)	−0.002 (3)	−0.016 (2)	0.003 (3)
C11	0.090 (3)	0.0654 (17)	0.082 (2)	0.004 (2)	−0.013 (2)	0.0145 (17)
C12	0.101 (3)	0.078 (2)	0.099 (3)	0.025 (2)	−0.005 (2)	−0.029 (2)
C13	0.047 (2)	0.0554 (16)	0.0464 (16)	−0.0003 (15)	−0.0049 (16)	−0.0037 (14)
C14	0.052 (2)	0.0640 (17)	0.0426 (18)	−0.0003 (15)	−0.0025 (15)	−0.0071 (14)
C15	0.052 (2)	0.0674 (19)	0.057 (2)	−0.0010 (17)	−0.0063 (18)	0.0047 (17)
C16	0.057 (2)	0.0535 (16)	0.0482 (17)	−0.0012 (15)	−0.0009 (17)	−0.0027 (14)
Cl1	0.1203 (10)	0.1131 (7)	0.0485 (5)	0.0028 (6)	0.0062 (5)	−0.0091 (4)
N1	0.065 (2)	0.0527 (13)	0.0646 (16)	0.0081 (13)	−0.0033 (15)	−0.0077 (12)
O1	0.0491 (14)	0.0721 (11)	0.0360 (10)	−0.0039 (10)	−0.0030 (10)	−0.0042 (9)
O2	0.0772 (17)	0.0524 (11)	0.0586 (14)	0.0124 (11)	−0.0053 (12)	−0.0035 (10)
O3	0.0903 (19)	0.0725 (13)	0.0823 (16)	0.0021 (13)	−0.0102 (15)	0.0247 (12)

### Geometric parameters (Å, °)

**Table:**

C1—C6	1.502 (5)	C9—H9A	0.9600
C1—C2	1.544 (4)	C9—H9B	0.9600
C1—H1A	0.9700	C9—H9C	0.9600
C1—H1B	0.9700	C10—H10A	0.9600
C2—C3	1.521 (4)	C10—H10B	0.9600
C2—C8	1.536 (5)	C10—H10C	0.9600
C2—H2	0.9800	C11—N1	1.453 (3)
C3—O1	1.447 (3)	C11—H11A	0.9600
C3—C4	1.511 (4)	C11—H11B	0.9600
C3—H3	0.9800	C11—H11C	0.9600
C4—C5	1.533 (4)	C12—N1	1.454 (4)
C4—H4A	0.9700	C12—H12A	0.9600
C4—H4B	0.9700	C12—H12B	0.9600
C5—C6	1.509 (6)	C12—H12C	0.9600
C5—C7	1.523 (5)	C13—N1	1.331 (3)
C5—H5	0.9800	C13—C14	1.354 (3)
C6—H6A	0.9700	C13—C16	1.518 (4)
C6—H6B	0.9700	C14—C15	1.428 (4)
C7—H7A	0.9600	C14—Cl1	1.713 (3)
C7—H7B	0.9600	C15—O3	1.205 (3)
C7—H7C	0.9600	C15—O2	1.373 (3)
C8—C9	1.521 (4)	C16—O1	1.387 (3)
C8—C10	1.541 (5)	C16—O2	1.434 (3)
C8—H8	0.9800	C16—H16	0.9800
			
C6—C1—C2	112.7 (3)	C2—C8—H8	106.8
C6—C1—H1A	109.0	C10—C8—H8	106.8
C2—C1—H1A	109.0	C8—C9—H9A	109.5
C6—C1—H1B	109.0	C8—C9—H9B	109.5
C2—C1—H1B	109.0	H9A—C9—H9B	109.5
H1A—C1—H1B	107.8	C8—C9—H9C	109.5
C3—C2—C8	114.2 (3)	H9A—C9—H9C	109.5
C3—C2—C1	108.8 (3)	H9B—C9—H9C	109.5
C8—C2—C1	113.7 (3)	C8—C10—H10A	109.5
C3—C2—H2	106.5	C8—C10—H10B	109.5
C8—C2—H2	106.5	H10A—C10—H10B	109.5
C1—C2—H2	106.5	C8—C10—H10C	109.5
O1—C3—C4	112.3 (2)	H10A—C10—H10C	109.5
O1—C3—C2	105.8 (2)	H10B—C10—H10C	109.5
C4—C3—C2	111.7 (3)	N1—C11—H11A	109.5
O1—C3—H3	109.0	N1—C11—H11B	109.5
C4—C3—H3	109.0	H11A—C11—H11B	109.5
C2—C3—H3	109.0	N1—C11—H11C	109.5
C3—C4—C5	112.1 (2)	H11A—C11—H11C	109.5
C3—C4—H4A	109.2	H11B—C11—H11C	109.5
C5—C4—H4A	109.2	N1—C12—H12A	109.5
C3—C4—H4B	109.2	N1—C12—H12B	109.5
C5—C4—H4B	109.2	H12A—C12—H12B	109.5
H4A—C4—H4B	107.9	N1—C12—H12C	109.5
C6—C5—C7	113.5 (3)	H12A—C12—H12C	109.5
C6—C5—C4	108.8 (3)	H12B—C12—H12C	109.5
C7—C5—C4	111.2 (3)	N1—C13—C14	132.7 (3)
C6—C5—H5	107.7	N1—C13—C16	120.7 (2)
C7—C5—H5	107.7	C14—C13—C16	106.5 (2)
C4—C5—H5	107.7	C13—C14—C15	110.3 (2)
C1—C6—C5	112.2 (3)	C13—C14—Cl1	131.5 (2)
C1—C6—H6A	109.2	C15—C14—Cl1	118.2 (2)
C5—C6—H6A	109.2	O3—C15—O2	120.4 (3)
C1—C6—H6B	109.2	O3—C15—C14	130.7 (3)
C5—C6—H6B	109.2	O2—C15—C14	108.9 (2)
H6A—C6—H6B	107.9	O1—C16—O2	110.2 (2)
C5—C7—H7A	109.5	O1—C16—C13	108.4 (2)
C5—C7—H7B	109.5	O2—C16—C13	105.1 (2)
H7A—C7—H7B	109.5	O1—C16—H16	111.0
C5—C7—H7C	109.5	O2—C16—H16	111.0
H7A—C7—H7C	109.5	C13—C16—H16	111.0
H7B—C7—H7C	109.5	C13—N1—C11	123.0 (2)
C9—C8—C2	114.1 (3)	C13—N1—C12	121.6 (3)
C9—C8—C10	110.4 (3)	C11—N1—C12	115.4 (2)
C2—C8—C10	111.6 (3)	C16—O1—C3	116.8 (2)
C9—C8—H8	106.8	C15—O2—C16	109.0 (2)
